# V101L of human formyl peptide receptor 1 (FPR1) increases receptor affinity and augments the antagonism mediated by cyclosporins

**DOI:** 10.1042/BJ20121839

**Published:** 2013-03-28

**Authors:** Caihong Zhou, Yan Zhou, Jia Wang, Yang Feng, Haonan Wang, Jinglun Xue, Yani Chen, Richard D. Ye, Ming-Wei Wang

**Affiliations:** *The National Center for Drug Screening, the Chinese Academy of Sciences Key Laboratory of Receptor Research and the State Key Laboratory of Drug Research, Shanghai Institute of Materia Medica, Chinese Academy of Sciences (CAS), Shanghai 201203, China; †Institute of Genetics, Fudan University, Shanghai 200433, China; ‡Shanghai ADICON Clinical Laboratories, Shanghai 200237, China; §School of Pharmacy, Shanghai Jiaotong University, Shanghai 200240, China

**Keywords:** cyclosporin, formyl peptide receptor 1 (FPR1), haplotype, pharmacogenomics, receptor affinity, single nucleotide polymorphism, CHO, Chinese-hamster ovary, CsA, cyclosporin A, CsH, cyclosporin H, ERK, extracellular-signal-regulated kinase, Fluo-4/AM, Fluo-4 acetoxymethyl ester, fMLF, *N*-formyl-methionyl-leucyl-phenylalanine, fNLFNYK, *N*-formyl-Nle-Leu-Phe-Nle-Tyr-Lys, FPR, formyl peptide receptor, HBSS, Hanks balanced saline solution, LD, linkage disequilibrium, MAF, minor allele frequency, MAPK, mitogen-activated protein kinase, ORF, open reading frame, SNP, single nucleotide polymorphism

## Abstract

Genetic variation plays a major role in drug response variability. CsA (cyclosporin A), a widely used immunosuppressive agent, is a specific antagonist for FPR1 (formyl peptide receptor 1), which is an important G-protein-coupled chemoattractant receptor in the innate immune system. In order to study the variable responses of cyclosporins to different FPR1 mutants, we investigated the distribution of human FPR1 haplotypes among 209 healthy Han Chinese subjects. The haplotype pattern in Han Chinese were characterized on the basis of five SNPs (single nucleotide polymorphisms), including rs5030878 (p.T11I), rs2070745 (p.V101L), rs5030880 (p.R190W), rs1042229 (p.N192K) and rs867228 (p.A346E). Receptor binding affinity of cyclosporins to FPR1 haplotypes was assessed using *N*-formyl-Nle-Leu-Phe-Nle-Tyr-Lys–FITC in CHO-G_α16_ cells stably transfected with cDNAs encoding the top 12 FPR1 haplotypes in the Han Chinese. Variants of FPR1 carrying a single amino acid substitution of leucine for valine at position 101 (p.Leu^101^) displayed significantly higher p*K*_i_ values for CsA and CsH (cyclosporin H), indicative of an improved receptor affinity. The polymorphism of *FPR1* p.Leu^101^ also enhanced the inhibitory effects of cyclosporins on fMLF (*N*-formyl-methionyl-leucyl-phenylalanine)-induced activities, including calcium mobilization, cell chemotaxis and MAPK (mitogen-activated protein kinase) phosphorylation. These results point to a possible complication for clinical use of CsA in patients carrying the p.Leu^101^ allele of *FPR1*.

## INTRODUCTION

Pharmacogenomics is an emerging research field that focuses on the study of how genes modulate drug responses among individuals [1,2]. An abundant volume of literature in this area indicates that genetic variation plays a major role in drug response variability, which is involved in pharmacodynamics and pharmacokinetics of a drug. Such variations, including SNPs (single nucleotide polymorphisms), base insertions or deletions, copy-number variations and variable numbers of tandem repeats, can influence, and hence potentially predict, both the efficacy and toxicity of a drug. As generally used markers throughout the genome, SNPs may exist in individual genes in coding regions, introns and surrounding regions. The haplotypes of several SNPs provide greater statistical power to detect genes that are associated with disease traits and drug responses. Genetically distinct populations may differ in both the extent of the LD (linkage disequilibrium) of these SNPs and their haplotype frequencies, which reflect the population structure and human evolution [3].

For an individual, their genetic make-up exerts influences in how well a medication works and what side effects are likely to occur within the body. Small differences in DNA sequence between different population groups or families within a population group, building up over generations, can lead to different reactivity to therapeutic agents. Following the completion of the Human Genome Project (http://www.ornl.gov/sci/techresources/Human_Genome/home.shtml), the International HapMap Consortium (2003, 2005) and the 1000 Genomes Project Consortium (2010), breakthroughs in genomics have boosted the development and application of pharmacogenomics, which has a potential clinical value when deciding between multiple treatment options to maximize therapeutic benefits and to limit the risk of side effects for individuals or special populations [4]. The number of pharmacogenetic associations has steadily increased over the years. PharmGKB (http://www.pharmgkb.org), a pharmacogenomics knowledge resource that encompasses clinical information, including dosing guidelines, drug labels, potentially clinically actionable gene–drug associations and genotype–phenotype relationships, annotated over 2000 genes involved in drug responses. Approximately 10% of the labels for FDA (Food and Drug Administration)-approved drugs contain pharmacogenomic information (http://www.fda.gov/drugs/scienceresearch/researchareas/pharmacogenetics/ucm083378.htm) [2,5]. It is the ultimate goal of pharmacogenomics to use an understanding of drug–gene associations to devise personalized treatment strategies for any given medication.

Cyclosporins are cyclic undecapeptides produced by fungi. Among them, CsA (cyclosporin A) was initially identified as an immunosuppressant and has been widely used clinically to prevent organ rejection following transplantation in humans [6]. CsA is lipophilic, readily penetrates the plasma membrane and exerts its immunosuppressive action through binding to the cytosolic protein cyclophilin, which causes inhibition of the Ca^2+^ and calmodulin-dependent phosphatase calcineurin, thereby disrupting transcriptional induction of interleukin-2 and T-cell activation [7]. A variety of structural analogues of CsA are produced either by fungi or through chemical synthesis [8–11]. Among them, CsH (cyclosporin H) is an optical isomer of CsA that contains the amino acid residue *N*-methyl-D-valine at position 11 instead of *N*-methyl-L-valine seen in CsA. CsH lacks immunosuppressive activity and only weakly interferes with the multidrug-resistance protein P-glycoprotein (MDR1). Several lines of evidence have indicated that cyclosporins are antagonists for human FPR (formyl peptide receptor) 1 [12].

The human *FPR1* gene is located in chromosome 19q13.3-19q13.4, clustered with the genes of another two receptors in the same receptor family, FPR2/ALX and FPR3 [13]. It contains three exons, but the ORF (open reading frame), which encodes a protein of 350 amino acids, locates exclusively in the third exon. At least ten SNP loci, four synonymous and six non-synonymous, with the least abundant allele showing a frequency of 0.1 or more, have been identified in the human *FPR1* coding region. It is known that SNPs occur throughout the genome at a frequency of approximately 1 in 300–1000 bp [14,15]. It is quite unique for the human *FPR1* gene to have such a high frequency of polymorphism. When nucleotide diversity, which is defined as the number of nucleotide differences per site between two randomly chosen sequences from a population, was used to quantify genetic variability of human *FPR1*, the value of *FPR1* was approximately 3-fold higher compared with the mean value reported for other genes [16]. Furthermore, the number of *FPR1* variants varies markedly in different mammalian species, indicating differential gene expansion or extinction [13]. Among different human racial groups, the *FPR1* SNPs are also detected at variable levels, which may suggest that the *FPR1* gene has been subjected to strong natural selection throughout evolution [16].

The protein encoded by the human *FPR1* gene is a seven-membrane-span G-protein-coupled receptor in the rhodopsin family. When stimulated by chemoattractants, such as the bacterial peptide fMLF (*N*-formyl-methionyl-leucyl-phenylalanine), formyl peptides released by mitochondria of ruptured cells [17,18], neutrophil granule protein cathepsin G [19], virus-derived peptides HIV-T20, T21 and HCV (hepatitis C virus) nonstructural peptide 5A [20–22], FPR1 activates heterotrimeric G-proteins and downstream signalling molecules, leading to neutrophil chemotaxis, degranulation and superoxide production [13]. These activities are essential to the elimination of invading pathogens and damaged tissues, suggesting that FPR1 plays an important role in the innate immune system. Experimental data indicate that mice lacking the *Fpr1* gene are more susceptible to *Listeria monocytogenes* infection with an increased mortality rate [23]. The pharmacogenomic survey of human *FPR1* polymorphism provides some hints about the function of this gene. It was first reported in 1977 by Clark et al. [24] that defective chemotactic responses were observed in neutrophils from seven out of nine juvenile periodontitis patients. Since then, an association between aggressive periodontitis and defects in neutrophil responses to formyl peptides was documented from time to time, involving SNPs including p.F110S, p.C126W, p.R190W, p.N192K, c.−12915C>T, c.301G>C, c.546C>A and c.348T>C [25–28]. There are also some inconsistent reports about the association of *FPR1* c.32C>T with C-reactive protein. Recently, El Shamieh et al. [29] reported that *FPR1* c.32C>T interacted with age, and was associated with high blood pressure in healthy individuals under 45 years of age. Owing to the different allele and genotype frequencies in various populations, studies on *FPR1* haplotypes in different racial groups were also carried out. To date, more than 20 haplotypes have been reported [16,30], and the investigation by Gripentrog et al. [30] found that some of the FPR1 haplotypes are functionally distinct when activated by formyl peptides (e.g. fMFEDAVAWF) from other bacterial strains such as *Mycobacterium avium* rather than the *Escherichia coli*-derived fMLF. The different SNPs and haplotypes of FPR1 may respond differently to some special modulators of the receptor. However, no drug-response association with the *FPR1* gene polymorphism has been described so far.

We have previously demonstrated that both CsA and CsH are selective antagonists for human FPR1 [12]. They inhibited the uptake of fNLFNYK (*N*-formyl-Nle-Leu-Phe-Nle-Tyr-Lys)–fluorescein and [^3^H]fMLF binding to FPR1. In functional assays, both cyclosporins inhibited fMLF-stimulated degranulation, chemotaxis, calcium mobilization and phosphorylation of MAPKs [mitogen-activated protein kinases; ERK1/2 (extracellular-signal-regulated kinase)] and serine/threonine protein kinase Akt. In 2002, Loor et al. [9] established the structure–activity relationships of 59 cyclosporins for FPR1 inhibition *in vitro* using intact live cells. However, no data could be found relative to investigations on pharmacogenomics of cyclosporins at different FPR1 haplotypes, especially in special racial groups. In the present study, we studied the distribution of human FPR1 haplotypes in the Han Chinese population. The variability of biological responses elicited by cyclosporins to these FPR1 haplotypes was also evaluated.

## EXPERIMENTAL

### Subjects

Blood samples were collected by ADICON Clinical Laboratories (Shanghai, China) from 209 healthy Han ethnic volunteers (ages ranged from 18 to 88 years, including 78 males and 131 females) in accordance with the procedure approved by the institutional review board. The participants were all aware of and agreed upon the tests to be performed through execution of individual informed consent forms. The samples were studied and analysed for *FPR1* gene polymorphisms anonymously and the present study was conducted according to the principles expressed in the Declaration of Helsinki.

### Reagents

Cyclosporins were produced at the Fujian Institute of Microbiology (Fuzhou, China). Fluo-4/AM (Fluo-4 acetoxymethyl ester) and fNLFNYK–FITC were obtained from Molecular Probes. Probenecid and fMLF were the products of Sigma–Aldrich. The anti-phospho-ERK1/2 and anti-phospho-Akt (recognizing Ser^473^) antibodies were from Cell Signaling Technologies. All restriction enzymes, DNA polymerase and DNA ligation kits were purchased from TaKaRa Biotechnology. DNA purification after electrophoresis was done with the TIANgel Mini Purification kit (Tiangen Biotech). PCR products were purified with the AxyPrep PCR Clean-up kit (Axyegen). Site-directed mutagenesis kits were bought from Stratagene.

### Vectors and cell lines

The expression vector pGEN-IRES Neo was provided by AstraZeneca. The cDNA encoding human FPR1 (NCBI accession number NM_002029.3) was obtained from a human HL-60 granulocyte library [31]. The plasmid pUC18 was obtained from Tiangen Biotech.

The cell line CHO-G_α16_ (RD-HGA16) was purchased from Molecular Devices. It consists of a CHO (Chinese-hamster ovary) cell host and the stably transfected plasmid for the G_α16_ protein. Cells were routinely maintained in Ham's F-12 medium supplemented with 10% (v/v) fetal bovine serum (Invitrogen), 2 mM L-glutamine, 100 μg/ml streptomycin, 100 units/ml penicillin and 100 μg/ml hygromycin.

### Genotype and haplotype determination

Genomic DNA was isolated from the cellular component of 1 ml of whole blood with the TIANamp Blood DNA kit according to the manufacturer's instructions (Tiangen Biotech). The ORF of the *FPR1* gene was amplified with PCR using primers located upstream and downstream of exon 3. The forward primer was 5′-TTGCCCAACAGGTACAATAA-3′ and the reverse primer was 5′-ATTTCAGGCAAACTAGGATG-3′. The products were sequenced with the primers 5′-AGAACCACCGCACCGTGA-3′ and 5′-GGATGTTCCGGCTGTTGT-3′. Gene polymorphisms were determined with sequencing by Shanghai DNA BioTechnologies. Five polymorphisms in the *FPR1* gene, including rs5030878 (p.T11I), rs2070745 (p.V101L), rs5030880 (p.R190W), rs1042229 (p.N192K) and rs867228 (p.A346E), were further studied. The construction and population frequencies of haplotypes were calculated with the SHEsis software using its haplotype construction element, the algorithm of which is designed based on an improved expectation maximization method [32]. Analysis of Hardy–Weinberg equilibria was performed using the Pearson χ-squared test.

### Site-directed mutagenesis

Site-directed mutagenesis was carried out according to the manufacturer's instructions (Stratagene). The *FPR1* cDNA was amplified with PCR using the forward primer 5′-TCCGAATTCATGGAGACAAATTCCTCTCTC-3′ and the reverse primer 5′-GCTTCTAGATCACTTTGCCTGTAACGCCAC-3′. The fragment was subcloned into the pUC18 vector between the EcoRI and XbaI restriction sites to produce pUC18-FPR1, which is the template for mutation analysis. PCRs were performed as follows: 95°C for 1 min, then 95°C for 1 min, 55°C for 1 min and 65°C for 8 min for 30 cycles. Primers used for the site-directed mutagenesis are listed in Supplementary Table S1 (at http://www.biochemj.org/bj/451/bj4510245add.htm). The resultant *FPR1* cDNA inserts encoding different haplotypes were verified with sequencing and ligated into the mammalian expression vector pGEN-IRES Neo between EcoRI and XbaI restriction sites.

Transfection of FPR1 haplotype cDNAs into CHO-G_α16_ cells was carried out with the Gene Pulser Xcell Electroporation System from Bio-Rad Laboratories. Briefly, approximately 4×10^6^ cells were harvested and resuspended in 400 μl of RPMI 1640 medium containing 10 mM glucose and 0.1 mM dithiothreitol. Vectors containing the ORFs of the human *FPR1* gene (8 μg in a volume of 8 μl) were added to the cells. Following a 280 V and 20 ms pulse (squarewave), the cells were immediately returned to 10 ml of culture medium and incubated for 24 h. Stable clones expressing FPR1 haplotypes were selected after 10 days of culture under the selection of 600 μg/ml G418 and identified by FACScan analysis after binding of 20 nM of the fluorescent ligand fNLFNYK–FITC to the CHO cell transfectants for 45 min at 4°C. Transfectants with similar expression levels of different FPR1 haplotypes were further confirmed by FACScan analysis after labelling with FITC-conjugated anti-FPR1 antibodies according to the manufacturer's instructions (BD Biosciences).

### FACScan analysis

CHO cells were added to 3 mM KCl, 100 mM NaCl, 10 mM sodium phosphate, 1 mM Mg^2+^ and 1 mM Ca^2+^, pH 7.4, containing 5% fetal bovine serum and incubated at 4°C for 1 h with various concentrations of fNLFNYK–FITC. The mean fluorescence of the cells was determined after subtracting the readout of cells without fNLFNYK–FITC. The results were analysed by non-linear least squares analysis to determine *K*_d_ and *B*_max_ values. For assessment of compound binding affinities to FPR1 haplotypes, cells were incubated with the indicated concentrations of cyclosporins together with 1 nM fNLFNYK–FITC. *K*_i_ values of different FPR1 variants were determined by non-linear least squares analysis using the known *K*_d_ for fNLFNYK–FITC and the observed IC_50_ numbers of cyclosporins in each case. Data from at least two different experiments in duplicate were analysed.

### Calcium mobilization

A calcium mobilization assay was performed as described previously with minor modifications [33]. Briefly, CHO-G_α16_-FPR1 cells were plated on to 96-well plates at a density of 15000 cells in 100 μl of medium per well and incubated for 24 h. Cells were loaded with 5 μM Fluo-4/AM in HBSS (Hanks balanced saline solution) supplemented with 2.5 mM probenecid for 45 min and then washed twice with HBSS. After incubation with or without cyclosporins for 15 min, 1 nM fMLF was added followed by analysis of calcium mobilization using a FlexStation (Molecular Devices) with the excitation wavelength at 485 nm and the emission wavelength at 525 nm.

### Chemotaxis

The chemotaxis assay was performed as described previously [12] in a 48-well microchemotaxis chamber (NeuroProbe). Briefly, fMLF (10 nM, 30 μl) was placed in the lower chamber and CHO-G_α16_-FPR1 cells (50 μl at 1×10^6^ cells/ml) were pre-incubated with or without cyclosporins for 15 min and then loaded on to the upper chamber, which was separated from the lower chamber by a polycarbonate filter (pore size of 8 μm). Chemotaxis was allowed to proceed for 4 h at 37°C. After that, cells on the upper face of the filter were removed. The cells that adhered to the underside of the filter were fixed and stained with 0.1% Crystal Violet [in 20% (v/v) methanol] for 30 min. The integrated absorbance of stained cells from three random fields of each well (triplicates for each concentration) was quantified using an image analyser (Image-Pro Plus). The chemotaxis of CHO-G_α16_-FPR1 cells induced with 1 nM fMLF was set as 100%.

### ERK1/2 activation

Activation of the p44/p42 MAPKs (ERK1/2) was determined as described previously [12] with modest modifications. Briefly, cells were cultured in 24-well plates at 5×10^5^ cells/well for 24 h and serum starved for 2 h. They were pretreated with the indicated amounts of cyclosporins for 5 min before fMLF (10 nM) stimulation in HBSS/BSA. After 5 min of stimulation, the reaction was terminated by adding 50 μl of ice-cold SDS/PAGE loading buffer [15% (v/v) glycerol, 125 mM Tris/HCl, pH 6.8, 5 mM EDTA, 2% (w/v) SDS, 0.1% Bromophenol Blue and 1% (v/v) 2-mercaptoethanol]. Samples were transferred to microcentrifuge tubes and sonicated twice for 5 s each to disperse DNA contents. After boiling, samples were analysed by SDS/PAGE and Western blotting using anti-ERK1/2 and anti-phospho-ERK1/2 antibodies at a 1:1000 dilution. Horseradish peroxidase-conjugated anti-rabbit antibody (1:3000 dilution) was used as the secondary antibody. The resulting immunocomplex was visualized using the SuperSignal West Pico chemiluminescence kit (Pierce Biotechnology) according to the manufacturer's instructions. The relative intensities of the bands from the PVDF membrane (Millipore) were quantified using the ChemDoc MP System (Bio-Rad) and Image-Pro Plus software (MediaCybernetics).

### Data analysis

The distribution of genotypes for each polymorphism was assessed for deviation from the Hardy–Weinberg equilibrium using w2 tests (StatView). Data on CHO cell transfectants were analysed using GraphPad Prism software. Non-linear regression analyses were performed to generate dose–response curves to calculate IC_50_ values.

## RESULTS

### FPR1 polymorphisms in the Han Chinese

Using genomic DNA isolated from whole blood cells derived from 209 healthy Han Chinese subjects, we analysed the distribution of allele and genotype of the human *FPR1* gene in this population. We selected all non-synonymous SNPs that have a MAF (minor allele frequency) higher than 0.1 for human *FPR1* from the dbSNP database (http://www.ncbi.nlm.nih.gov/SNP/snp_ref.cgi?locusId=2357). Five SNPs met this requirement, namely, rs5030878 (p.T11I), rs2070745 (p.V101L), rs5030880 (p.R190W), rs1042229 (p.N192K) and rs867228 (p.A346E). The SNP p.T11I is located at the N-terminus of the FPR1 protein, whereas p.V101L resides near the transmembrane–extracellular interface [13]. SNPs p.R190W and p.N192K are located in the second extracellular loop of FPR1. p.A346E is near the intracellular C-terminus of the receptor.

Next, we examined the prevalence of genetic variants containing these five *FPR1* SNPs in all subjects, including allele and genotype frequencies (Tables 1 and 2). The MAFs for p.T11I (c.32T), p.V101L (c.301C), p.R190W (c.568T) and p.A346E (c.1037A) were 0.029, 0.45, 0.172 and 0.297 respectively. The MAF value for p.V101L (0.45) found in the present study is consistent with that (0.477) derived from the HapMap resource (NCBI assay ID ss24686736), but it is approximately 1.5-fold higher than that found in other two ethnic groups (0.292 in European and 0.258 in Yoruba African populations respectively; Supplementary Figure S1 at http://www.biochemj.org/bj/451/bj4510245add.htm).The frequency of homozygous p.Ile^11^ was 0.005, closely resembling the value in Chinese from metropolitan Denver and differing from that in Chinese from Beijing. Although the variant for p.N192K was generally believed to be c.576T>G, our results displayed three alleles for p.N192K, with a frequency of 0.514 for c.576T, 0.304 for c.576G and 0.182 for c.576C respectively. Alleles C and T at this position are synonymous, and both are translated into p.Asn^192^, resulting in a much lower genotype frequency of p.Lys^192^ than those documented in the HapMap (0.30 compared with the reported values of 0.48 for Han Chinese in Beijing and 0.52 for Chinese in metropolitan Denver; Table 1). The LD pattern of these *FPR1* SNPs was analysed with pair-wise Lewontin's D’ and correlation coefficient *r*^2^ (Supplementary Figure S2 at http://www.biochemj.org/bj/451/bj4510245add.htm). The two adjacent SNPs, p.R190W and p.N192K, exhibited relative strong LD compared with other pairs of SNPs [34].

**Table 1:** Allele frequencies in the Han Chinese studied Data are derived from direct sequencing of genomic DNA from 209 Han Chinese subjects.

Reference ID	SNP	Allele	Phenotype	Number	Frequency
rs5030878	T11I	c.32C	Thr^11^	406	0.971
		c.32T	Ile^11^	12	0.029
rs2070745	V101L	c.301G	Val^101^	230	0.550
		c.301C	Leu^101^	188	0.450
rs5030880	R190W	c.568A	Arg^190^	346	0.828
		c.568T	Trp^190^	72	0.172
rs1042229	N192K	c.576T	Asn^192^	215	0.514
		c.576G	Lys^192^	127	0.304
		c.576C	Asn^192^	76	0.182
rs867228	A346E	c.1037C	Ala^346^	294	0.703
		c.1037A	Glu^346^	124	0.297

**Table 2:** Genotype frequencies in the Han Chinese studied Data are derived from direct sequencing of genomic DNA from 209 Han Chinese subjects. Amino acids are in the single-letter code.

Reference ID	SNP	Genotype	Number	Frequency
rs5030878	T11I	T/T	198	0.947
		T/I	10	0.048
		I/I	1	0.005
rs2070745	V101L	V/V	62	0.297
		V/L	106	0.507
		L/L	41	0.196
rs5030880	R190W	R/R	144	0.689
		R/W	58	0.278
		W/W	7	0.033
rs1042229	N192K	N/N	98	0.469
		N/K	95	0.455
		K/K	16	0.077
rs867228	A346E	A/A	106	0.507
		A/E	82	0.392
		E/E	21	0.100

Haplotype frequencies in the Han Chinese were inferred from the genotype information with SHEsis, which is based on the PLCSEM (Partition-Ligation Combination-Subdivision Expectation Maximization) algorithm and is robust for efficient estimation of haplotypes constructed from large numbers of biallelic or multiallelic loci in diploid individuals [32]. As depicted in Table 3, nine *FPR1* haplotypes with a frequency higher than 0.03 were constructed and eight more were inferred in the Han Chinese. Haplotypes CH1–CH13 have all been reported previously [16,30]. However, the distribution of these haplotypes differs markedly from that reported in other races. As described in the literature [16,30], the top two haplotypes in the Caucasian population were CH3 (p.Lys^192^, H-1) and CH6 (p.Trp^190^, H-2), whereas the third one carrying p.Ile^11^/Lys^192^/Glu^346^ in the Caucasian population could not be detected in Han Chinese. CH14 (Ile^11^/Leu^101^/Glu^346^) is a newly discovered haplotype never reported before. These results demonstrate that the pattern and distribution of FPR1 haplotypes differ between Caucasian and Han Chinese populations, thus representing the different structure and evolution pathway of these two populations.

**Table 3:** Haplotype frequencies in the Han Chinese studied Amino acids are in the single-letter code.

		SNP					
Haplotype*	Original nomenclature†	I11T	V101L	R190W	N192K	A346E	Frequency (%)
CH1	H-6A	T	L	R	N-t‡	A	15.9
CH2	H-11	T	V	R	N-t	A	13.2
CH3	H-1	T	V	R	K	A	12.1
CH4	H-12	T	V	R	N-t	E	11.5
CH5	H-5	T	L	R	K	A	10.2
CH6	H-2	T	V	W	N-c	A	8.8
CH7	H-16	T	V	R	K	E	8.1
CH8	H-19	T	L	R	N-t	E	7.9
CH9	H-17	T	L	W	N-c	A	6.5
CH10	H-20	T	L	W	N-c	E	2.0
CH11	H-8	I	V	R	N-t	A	1.3
CH12	H-4	I	L	R	N-t	A	1.3
CH13	H-6B	T	L	R	N-c	A	1.0
CH14	-§	I	L	R	N-t	E	0.2
CH15	H-25A	T	L	R	K	E	0
CH16	H-21	T	V	W	N-c	E	0
CH17	H-18	I	V	W	N-c	A	0

*Each haplotype is defined by the shown linkage of nucleotides in the corresponding row, and each corresponds to the SNPs listed in the top row. Haplotypes were determined with the SHEsis software based on the genotype information from sequencing.

†Original nomenclature follows that of [7,21], and the number represents the ranking of haplotypes in the Caucasian population.

‡SNP that does not change the amino acid.

§Newly discovered haplotype that has not been reported previously.

### Binding affinity of cyclosporins to FPR1 mutants

CsA is an immunosuppressant widely used in the clinic for patients receiving organ transplantation. In order to study whether the polymorphism of the human *FPR1* gene affects the pharmacology of cyclosporins, especially in the Han Chinese, we constructed a batch of *FPR1* cDNAs encoding the first 12 FPR1 haplotypes in this population. Vectors including *FPR1* ORFs were transfected into CHO-G_α16_ cells and stable cell lines expressing similar levels of individual FPR1 haplotypes were selected. The expression of the FPR1 haplotype in each clone was determined by FACScan analysis using the peptide fNLFNYK–FITC as a probe, which was subsequently confirmed with FITC-conjugated anti-FPR1 antibodies. As shown in Table 4, the *B*_max_ values of CHO-G_α16_-FPR1 clones containing different FPR1 haplotypes were in the range of 33860 in CH4 (p.Glu^346^)-expressing cells (CH4) to 65059 in CH12 (p.Ile^11^/Leu^101^)-expressing cells (CH12). The receptor affinity of fNLFNYK–FITC to different FPR1 haplotypes varied moderately. Compared with CH1 (p.Leu^101^), haplotypes CH5 (p.Leu^101^/Lys^192^) and CH12 (p.Ile^11^/Leu^101^) had the highest affinity (*K*_d_=2.8±0.1 and 3.2±0.3 nM respectively), whereas haplotypes CH4 (p.Glu^346^) and CH8 (p.Leu^101^/Glu^346^) showed the lowest affinity (*K*_d_=1.3±0.2 and 1.3±0.1 nM respectively).

**Table 4:** Receptor-binding affinity of cyclosporins to FPR1 haplotypes

	fNLFNYK–FITC-binding parameters*		p*K*_i_ for cyclosporins†	
Haplotype	*K*_d_ (nM)	*B*_max_	p*K*_i_ for CsA	p*K*_i_ for CsH
CH1	2.0±0.2	48336±4553	7.15±0.05	8.16±0.16
CH2	1.7±0.1	47657±9098	5.26±0.02‡	6.34±0.04‡
CH3	1.8±0.2	59135±4185	5.62±0.09‡	6.48±0.04‡
CH4	1.3±0.2‡	33860±4977	5.99±0.12‡	6.16±0.08‡
CH5	2.8±0.1‡	42575±4902	6.86±0.08	7.75±0.09
CH6	1.8±0.4	34093±693	5.63±0.02‡	6.46±0.05‡
CH7	1.7±0.3	60688±10388	5.85±0.02‡	6.47±0.04‡
CH8	1.3±0.1‡	36362±353	6.96±0.11	7.90±0.08
CH9	1.9±0.5	39009±6672	7.05±0.09	7.91±0.11
CH10	2.4±0.3	41751±615	7.13±0.12	7.94±0.06
CH11	2.2±0.7	37713±5841	5.59±0.01‡	6.27±0.02‡
CH12	3.2±0.3‡	65059±3570	7.26±0.02	7.91±0.01

*CHO-G_α16_ cells stably transfected with individual variants of *FPR1* (CH1–CH12) were incubated with various concentrations of fNLFNYK–FITC. The data were analysed by non-linear least-squares analysis to determine *K*_d_ and *B*_max_ values.

†Cells were incubated with various concentrations of cyclosporins together with 1 nM fNLFNYK–FITC. *K*_i_ was determined by non-linear least-squares analysis using the known *K*_d_ for fNLFNYK–FITC and the observed IC_50_ of cyclosporins for different FPR1 variants.

‡*P*<0.05 compared with CH1.

**Figure 1:**
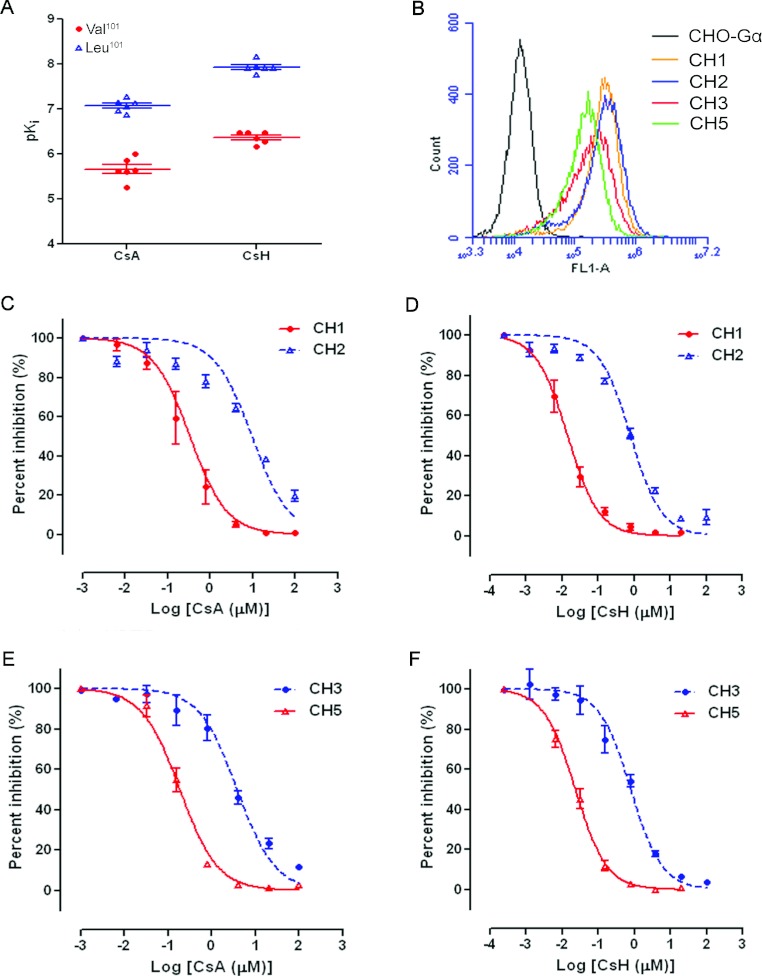
Effects of *FPR1* gene polymorphisms on the receptor-binding affinity for CsA and CsH (**A**) Distribution of p*K*_i_ values for CsA and CsH in CHO-G_α16_-FPR1 haplotype-expressing cells. (**B**) Flow cytometric analysis of cell-surface expression of FPR1 in control CHO-G_α16_ cells and haplotype CH1, CH2, CH3 or CH5-expressing CHO-G_α16_-FPR1 cells. (**C**–**F**) Concentration–response data of CH1, CH2, CH3 and CH5 cells to CsA and CsH were analysed. fNLFNYK–FITC (1 nM) was used as an agonist, and the value of agonist alone was set as 100%. Data are presented as means±S.E.M. derived from two to three independent experiments.

The binding affinity of CsA and CsH to mutant FPR1 receptors was also assessed in a competitive binding assay using 1 nM fNLFNYK–FITC (Table 4). We observed that the binding affinity of cyclosporins to different FPR1 mutants varied significantly. Haplotypes CH1, CH5, CH8, CH9, CH10 and CH12 all showed decreased *K*_i_ values for both CsA and CsH. These variants share a common allele, p.Leu^101^ (Figure 1A), indicating that p.Leu^101^ is a major factor that contributes variations to ligand binding of the receptor. The average *K*_i_ values of FPR1 for CsA and CsH increased in Leu^101^-expressing cells by 25.9- and 36.8-fold respectively. No other SNP demonstrated significant and consistent variation between these haplotypes. In order to determine the influence of amino acid variation of p.V101L on FPR1 affinity for cyclosporins, pairs of haplotypes containing only one variation at position 101, including CH1 (p.Leu^101^) compared with CH2 and CH3 (p.Lys^192^) compared with CH5 (p.Leu^101^/Lys^192^), were selected for further analysis. When assessed with anti-FPR1 antibodies, the surface expression profile of FPR1 on the cells expressing these haplotypes was comparable between each of the pairs tested (Figure 1B), but their affinity for CsA and CsH varied. The p*K*_i_ value of CsA increased from 5.26±0.02 in CH2-expressing cells to 7.15±0.05 in CH1-expressing cells, whereas those of CsH improved from 6.34±0.04 in CH2 to 8.16±0.16 in CH1 (Table 4, Figures 1C and 1D). The results for the haplotype pair CH3 and CH5 displayed a similar tendency: the p*K*_i_ value of CsA increased from 5.62±0.09 in CH3-expressing cells to 6.86±0.08 in CH5-expressing cells (Figure 1E). In this pair, the binding affinity of CsH also improved significantly (p*K*_i_=6.48±0.04 in CH3 compared with p*K*_i_=7.75±0.09 in CH5; Figure 1F).

### Enhanced antagonism of cyclosporins on calcium mobilization in FPR1 p.Leu^101^ mutants

**Figure 2:**
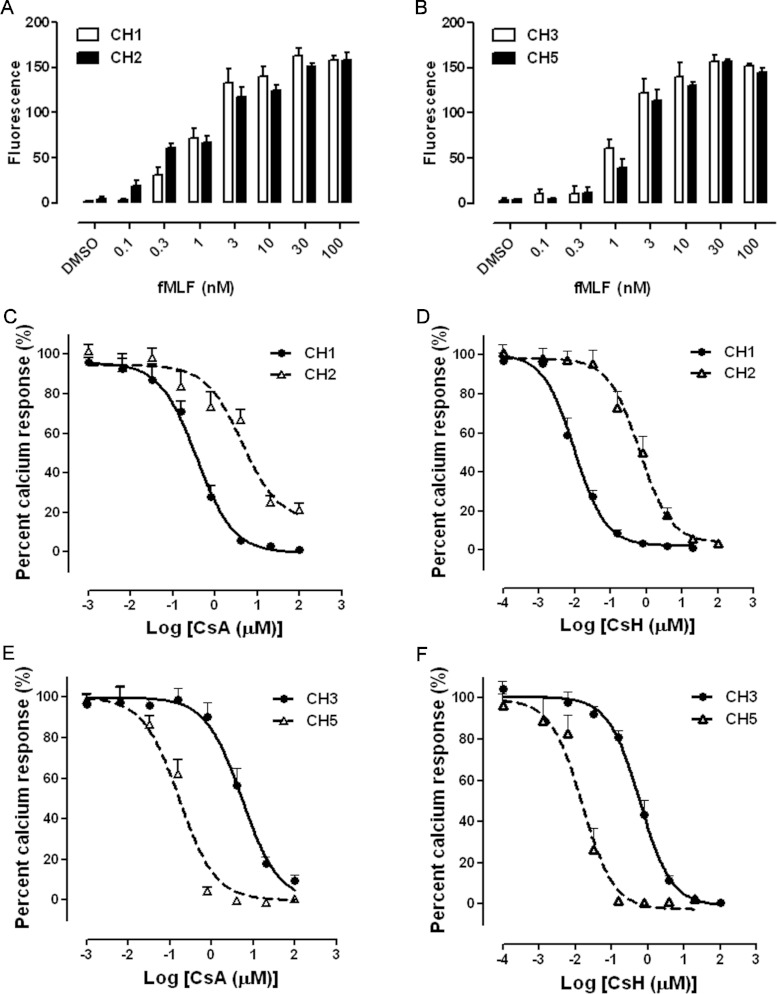
Inhibitory effects of cyclosporins on fMLF-mediated calcium mobilization in CHO-G_α16_-FPR1 haplotype-expressing cells Different concentrations of fMLF-induced calcium mobilization in CH1 and CH2 cells (**A**) and in CH3 and CH5 cells (**B**) are shown. (**C**–**F**) Cells were cultured overnight, loaded with 5 μM Fluo-4/AM and incubated with the indicated concentrations of CsA or CsH for 15 min before stimulation with 1 nM fMLF. Calcium mobilization was recorded on a FlexStation^III^. The fluorescence signal stimulated with fMLF alone was arbitrarily set as 100%. Data are presented as means±S.E.M. derived from at least three independent experiments.

Our previous study elucidated that both CsA and CsH affect the signal transduction and cellular function of FPR1, including calcium mobilization, degranulation and chemotaxis [12]. In order to investigate whether the substitution of leucine for valine at position 101 of FPR1 would affect the pharmacological property of cyclosporins at the receptor, calcium mobilization induced by fMLF in haplotype-expressing CHO-G_α16_-FPR1 cells was carried out. The peptide fMLF elicited calcium mobilization dose-dependently in the four cell lines tested (Figures 2A and 2B). When CsA was added 15 min before stimulation with 1 nM fMLF, its IC_50_ decreased greatly from 4.35 μM in CH2 to 0.36 μM in CH1 and from 5.29 μM in CH3 to 0.17 μM in CH5 (Figures 2C and 2E). Upon the addition of CsH, IC_50_ values in FPR1 p.Leu^101^-expressing cells were also markedly lower than those in FPR1 p.Val^101^-expressing cells (9.9 nM in CH1 compared with 647 nM in CH2, and 15.5 nM in CH5 compared with 605 nM in CH3) (Figures 2D and 2F).

### Enhanced antagonism of cyclosporins on chemotaxis in FPR1 p.Leu^101^ mutants

**Figure 3:**
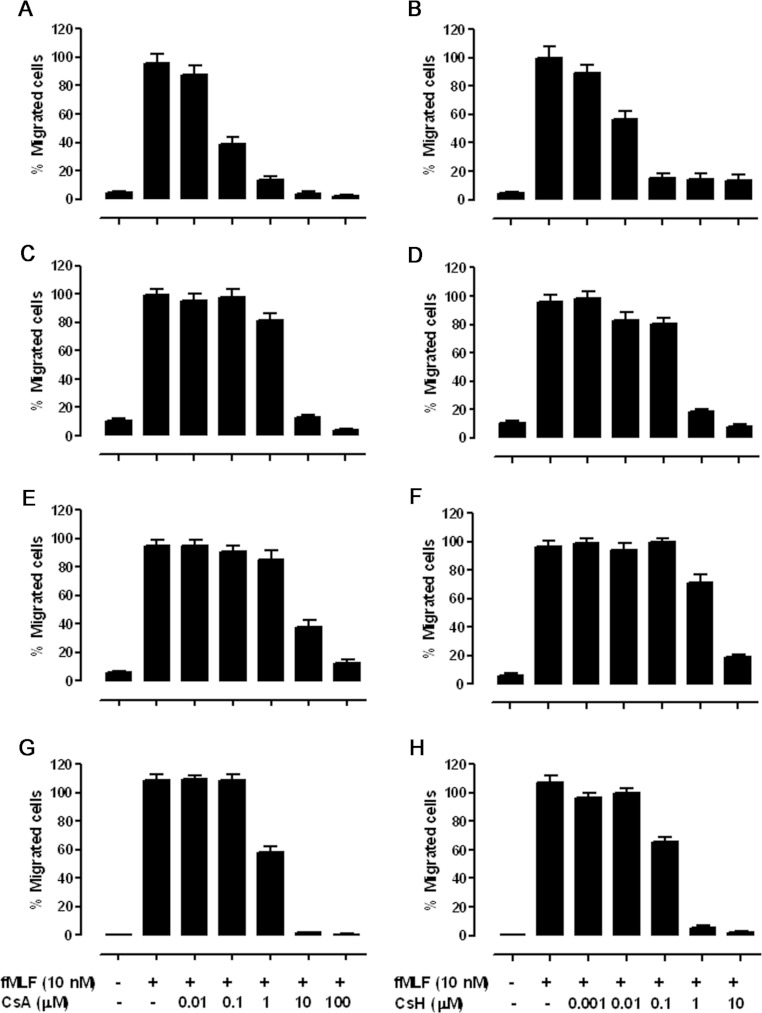
Effects of cyclosporins on fMLF-stimulated chemotaxis in FPR1 haplotype-expressing CHO-G_α16_ cells (**A** and **B**) CH1, (**C** and **D**) CH2, (**E** and **F**) CH3 and (**G** and **H**) CH5. Cells were pre-incubated with different concentrations of cyclosporins for 15 min before loading into the upper wells of a 48-well chemotaxis chamber with 10 nM fMLF placed in the lower wells. The chemotaxis assay was conducted at 37°C for 4 h, and the numbers of migrated cells were determined by analysis with the Image-Pro Plus software, i.e. counting the integrated absorbance of cells after staining with 0.01% Crystal Violet in 20% methanol. Three fields were photographed for each well. The results presented are means±S.E.M. from three independent experiments, each in triplicate, showing the percentage of maximal chemotaxis induced with fMLF alone.

Chemotaxis towards bacterial formylated peptides is one of the primary functions of FPR1. Besides the influence of *FPR1* gene polymorphism on fMLF-mediated calcium mobilization, we also monitored the inhibitory effects of CsA and CsH on cell chemotaxis induced by fMLF in CHO-G_α16_-FPR1 cells. The CHO transfectants with different FPR1 haplotypes were allowed to migrate for 4 h across a polycarbonate filter with 8 μm pores towards a lower well containing 10 nM fMLF. The inhibition on chemotaxis was greatly enhanced when p.Val^101^ of FPR1 was changed to p.Leu^101^, as depicted in Figure 3. In CH2 cells, the IC_50_ of CsA was 3.07 μM, whereas that of CsH was 320 nM. In CH1 cells, the inhibitory effects on chemotaxis were evident at CsA concentrations above 10 nM (IC_50_=61.8 nM) and at CsH concentrations above 1 nM (IC_50_=9.7 nM). Likewise, CsA inhibited fMLF-induced chemotaxis with a potency approximately 5-fold lower in CH5 (IC_50_=1.16 μM) than in CH3 (IC_50_=5.86 μM) cells, whereas the antagonism exerted by CsH was more significant between them (with IC_50_ values of 2.87 μM for CH3 compared with 165.1 nM for CH5 cells).

### Improved inhibition of cyclosporins on ERK1/2 activation in FPR1 p.Leu^101^ mutants

**Figure 4:**
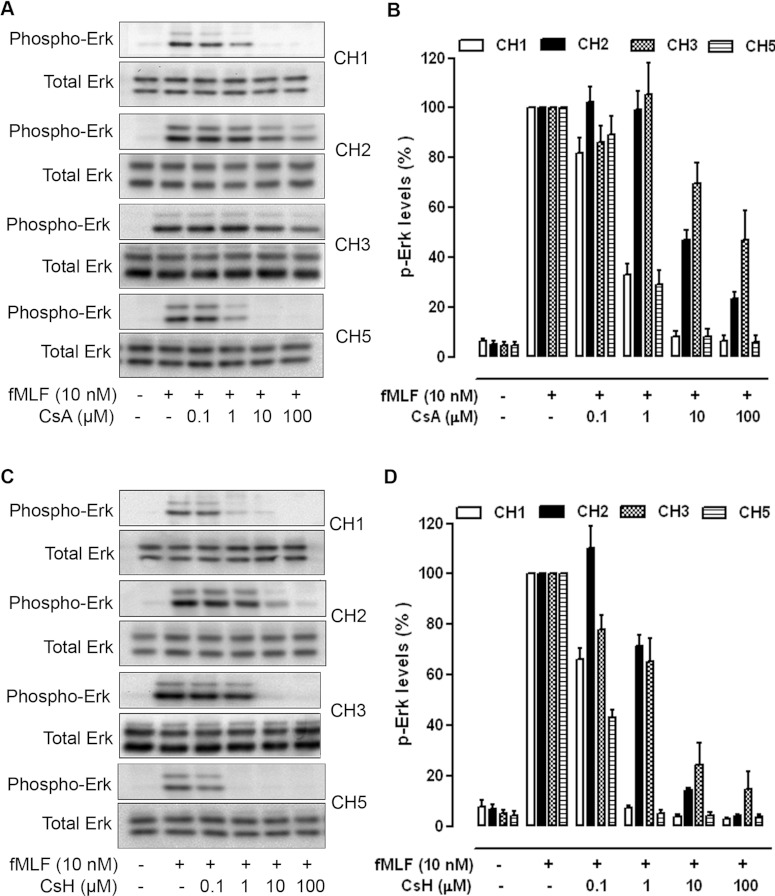
Inhibition of ERK1/2 phosphorylation by CsA and CsH CHO-G_α16_-FPR1 cells were treated with either CsA (**A** and **B**) or CsH (**C** and **D**) for 15 min as indicated and stimulated with fMLF (10 nM) for 5 min. Cell lysates were prepared, separated by SDS/PAGE and blotted with specific anti-phospho-ERK1/2 antibodies as described in the Experimental section. The total protein kinase levels in cell lysates were determined by blotting with antibodies against the non-phosphorylated ERK1/2, as shown at the bottom of each panel. Images displayed in (**A**) and (**C**) are representative of at least three independent experiments with similar results. The results in (**B**) and (**D**) are the means±S.E.M. of three independent experiments, with the ERK1/2 phosphorylation induced by fMLF set as 100%.

fMLF-induced FPR1 activation signals through heterotrimeric G-proteins and stimulates downstream kinases such as ERK1/2 and Akt. Since phosphorylation of ERK1/2 and Akt are early signalling events triggered by chemoattractants in leucocytes, we performed experiments to evaluate whether variation in the *FPR1* gene polymorphism p.V101L affects ERK1/2 phosphorylation. In CHO-G_α16_-FPR1 cells, fMLF (10 nM) elicited a significant increase in the phosphorylation of ERK1/2 (Figure 4). Treatment of the cells with CsA or CsH prior to fMLF stimulation concentration-dependently inhibited phosphorylation of the kinases. In this assay, CsH is approximately 10-fold more potent than CsA on the basis of the level of protein phosphorylation. The variants carrying p.Leu^101^ displayed a marked inhibition of fMLF-stimulated ERK1/2 phosphorylation at CsA and CsH concentrations above 1 μM and 100 nM respectively. In cells expressing FPR1 p.Val^101^, the potency of CsA and CsH in suppressing fMLF-induced ERK1/2 phosphorylation was approximately 10-fold lower than those in FPR1 p.Leu^101^-expressing cells.

## DISCUSSION

In the present study, we investigated the distribution of human FPR1 haplotypes in Han Chinese subjects using five SNPs, p.T11I, p.V101L, p.R190W, p.N192K and p.A346E. Stable cell lines expressing the top 12 FPR1 haplotypes in the Han Chinese were developed in CHO-G_α16_ cells and binding affinities of CsA and CsH to these FPR1 variants were evaluated. The results of the present study indicate that FPR1 haplotypes carrying a single amino acid substitution of leucine for valine at position 101 possess significantly higher affinities for CsA and CsH (Figure 1A and Table 4), thereby inhibiting fMLF-induced calcium mobilization, chemotaxis and MAPK phosphorylation in a more potent manner. The present study is among the first to investigate variable responses of FPR1 haplotypes to cyclosporins and to characterize the effects of *FPR1* gene polymorphisms such as that in the Han Chinese population.

SNPs are the simplest form of DNA variation among individuals, but covers approximately 80% of genome variations. Most SNPs in modern humans probably arose by single base-modifying events that took place a long time ago, which at the instance of creation would have been surrounded by a series of alleles at polymorphic loci and transmitted together. A unique grouping of alleles was established to be a haplotype. Thus haplotype is a more discriminative state of a chromosomal region and the basis for linkage analysis, which is very valuable for studying the genetics behind common diseases, genetic demography and chromosomal evolution [14]. For this purpose, haplotype has been investigated extensively in the human species by the International HapMap Project. In the present study we characterized the FPR1 haplotype pattern in the Han Chinese using five SNPs, p.T11I [29,35], p.V101L [25,27], p.R190W [25], p.N192K [25,28] and p.A346E, most of which were previously reported to be associated with the activation, downstream signal transduction and disease association of FPR1. When comparing with the HapMap data, we found that all five polymorphisms were common in the three racial groups, but the allele frequencies varied markedly among different racial groups, especially for alleles p.Ile^11^ and p.Leu^101^ (Supplementary Figure S1). In the Han Chinese, the frequency of p.Ile^11^ was less than a half of the frequencies seen in Caucasian and Yoruba populations. The frequency of p.Leu^101^ in the present study is 0.45 in the Han Chinese, approximately 1.5-fold higher than that reported in the other two ethnic groups. We also identified a third allele for p.N192K, and the resultant minor allele frequency of p.Lys^192^ was much lower than that documented in the HapMap database. On the basis of this information, we constructed nine FPR1 haplotypes with frequencies higher than 3% and inferred eight more in the Han Chinese (Table 3). Except for the newly identified haplotype 14, which carries FPR1 p.Ile^11^/Leu^101^/Glu^346^, all other haplotypes exist in both Han Chinese and Caucasian populations with considerable frequency variations.

The regional distribution of haplotypes will reflect not only the biological processes such as mutation and recombination, but also population-specific demographic history, such as bottlenecks, admixture, inbreeding, migration, immigration and assortative mating. Due to its high and variable levels of polymorphism in different racial groups, human *FPR1* is a good case to study the evolution of the ethnic population. Sahagun-Ruiz et al. [16] once compared the differences among primate haplotypes at sites that are polymorphic in human *FPR1*, and deduced that the presence of these polymorphisms in all racial groups tested argues against the possibility of a population bottleneck, but in favour of natural selection. FPR1 is known to be important in the chemotaxis of neutrophils and monocytes in response to bacterium-derived N-formyl peptides. Thus it is conceivable that infection of epidemic bacterial plagues, such as cholera, salmonella, bubonic plague, tuberculosis and pertussis might have created selective pressure on the evolution of the *FPR1* gene.

Whether different patterns of haplotypes affect FPR1 expression or function in primary cells remains unknown. The high frequency of *FPR1* polymorphic alleles implies that they may be suitable to disease association and drug response studies. Obviously, the highly divergent haplotype patterns for human FPR1 point to the potential of developing therapeutic strategies to target different ethnic populations. Our previous work has established that both CsA and CsH interact with FPR1 through cognitive binding to the receptor [12]. Inhibition by CsA and CsH on fMLF-induced FPR1 activities affects downstream events such as calcium mobilization, chemotaxis, degranulation, MAPK and Akt activation. However, it is still unclear how CsA and CsH interact with FPR1 and what structural basis dictates the different potency between CsA and CsH in FPR1 antagonism. Previous site-directed mutagenesis of the *FPR1* gene and structure–activity relationship studies on cyclosporins revealed some key sites that may be important for the formation of a binding pocket of FPR1. Loor et al. [9] reported that cyclosporins might bind to a single pharmacophore on FPR1. The latter normally maintains itself in an inactive conformation by an ion pair between Lys^85^ in the second transmembrane α-helix and Asp^284^ in the seventh transmembrane α-helix near the transmembrane–extracellular interface. It is believed that binding of agonists activates the receptor by disrupting such pairing or recruiting FPR1 conformers with a disrupted ion pair. Cyclosporins could compete with the binding of agonists to FPR1, and their interaction with FPR1 might also locate at the same region. Our experimental data indicate that Val^101^ of FPR1 may be a part of the binding site for cyclosporins. Val^101^ locates at the interface between the second transmembrane domain and the first extracellular loop, proximal to the predicted disulfide bond between Cys^98^ and Cys^176^ [13]. Compared with p.Val^101^, variants of FPR1 carrying p.Leu^101^ displayed significantly improved receptor affinity, thereby enhancing the inhibition of cyclosporins on FPR1-mediated functions, including calcium mobilization, chemotaxis and MAPK phosphorylation. Both CsA and CsH demonstrated the same trend, but questions remain as to why Leu^101^ could enhance the antagonism of cyclosporins on FPR1 so dramatically. Clearly, it would be of interest to learn whether cyclosporins could differentially inhibit fMLF-mediated superoxide generation and degranulation among different FPR1 variants. Functional FPR1 was initially identified and is mainly detectable in phagocytic leucocytes. Although cells of non-haemopoietic origin (e.g. epithelial cells and hepatocytes) also express this receptor, the associated biological significance has yet to be defined [13,36]. Using cells that express endogenous FPR1 (e.g. neutrophils) may be helpful in the conduction of functional assays for the variants.

A primary aim of pharmacogenomics is to identify novel human genetic variants responsible for phenotypic differences in drug efficacies. Biomarkers applicable to pharmacogenomics are becoming a major player in personalized medicine [5]. The success of this approach will depend upon having accurate diagnostic tests that identify patients who can benefit from targeted therapies while minimizing the risk of adverse effects. In the clinic, most drugs are often administered at maximally tolerated doses, which are typically based on population averages, resulting in unnecessary toxicity in some population and poor or no response in the other. As a widely used immunosuppressant, concerns remain relative to the therapeutic window of CsA. Although CsA achieves its therapeutic effects through signalling pathways unrelated to FPR1, over-suppression of FPR1 function by CsA may result in side effects that are not fully recognized. The most important effect of CsA is to lower the activity of T-cells and their immune responses through binding to the cytosolic protein cyclophilin (immunophilin). It also inhibits lymphokine production and interleukin release, leading to reduced functions of effector T-cells. This implies that in patients undergoing CsA therapy, immune responses mediated by T-cells are weakened. Therefore it is not unreasonable to deduce that CsA recipients who carry p.Leu^101^ variants of FPR1 may be more susceptible to pathogen infection than those displaying the p.Val^101^ phenotype, and this is especially noteworthy in a population with a relatively high p.Leu^101^ allele frequency such as the Han Chinese.

In conclusion, the results of the present study suggest that haplotypic variation in FPR1, especially the SNP p.V101L, alters the receptor's response to cyclosporins, which may have implications in guiding the clinical application of CsA. In order to translate this discovery into bedside practices, further work is necessary to replicate the findings in subjects carrying this variant in a larger population scale.

## Online data

Supplementary data
