# Supplementary material for: V101L of human formyl peptide receptor 1 (FPR1) increases receptor affinity and augments the antagonism mediated by cyclosporins
Source: Biochem J. 2013 Mar 28;451(Pt 2):245–55. doi: 10.1042/BJ20121839 (PMC3632010; doi:10.1042/BJ20121839)
Supplement: Supplementary data [file bj4510245add.pdf]

Biochem. J. (2013) **451**, 245–255 (Printed in Great Britain) doi:10.1042/BJ20121839

SUPPLEMENTARY ONLINE DATA

V101L of human formyl peptide receptor 1 (FPR1) increases receptor affinity and augments the antagonism mediated by cyclosporins

Caihong ZHOU*†, Yan ZHOU*, Jia WANG*, Yang FENG*, Haonan WANG*, Jinglun XUE†, Yani CHEN‡, Richard D. YE§ and Ming-Wei WANG*[1]

*The National Center for Drug Screening, the Chinese Academy of Sciences Key Laboratory of Receptor Research and the State Key Laboratory of Drug Research, Shanghai Institute of Materia Medica, Chinese Academy of Sciences (CAS), Shanghai 201203, China, †Institute of Genetics, Fudan University, Shanghai 200433, China, ‡Shanghai ADICON Clinical Laboratories, Shanghai 200237, China, and §School of Pharmacy, Shanghai Jiaotong University, Shanghai 200240, China

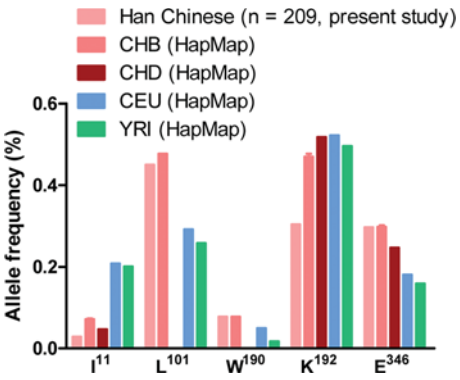

Figure S1 Allele frequencies of polymorphic amino acid positions in FPR1 from different racial groups

Amino acids are in the single-letter code. The results are combined from the present study and that documented in the HapMap database. CHB, Han Chinese in Beijing, China; CHD, Chinese in metropolitan Denver, Colorado; CEU, Utah residents with Northern and Western European ancestry from the CEPH collection; YRI, Yoruba in Ibadan, Nigeria.

Table S1 Primers used in the present study

Reference ID	SNP	Primer sequence
rs5030878	T11I	5′-tctccccacgaacacctctggagggacac-3′
rs2070745	V101L	5′-ggttcctgtgcaaattcctctttaccatagtggac-3′
rs5030880	R190W	5′-ccaacgaccctaaagagtggataaatgtggccgtt-3′
rs1042229	N192K	5′-acgaccctaaagagaggataaaggtggccgttgcc-3′
rs867228	A346E	5′-ccttctgcagaggtggagttacaggcaaagtga-3′

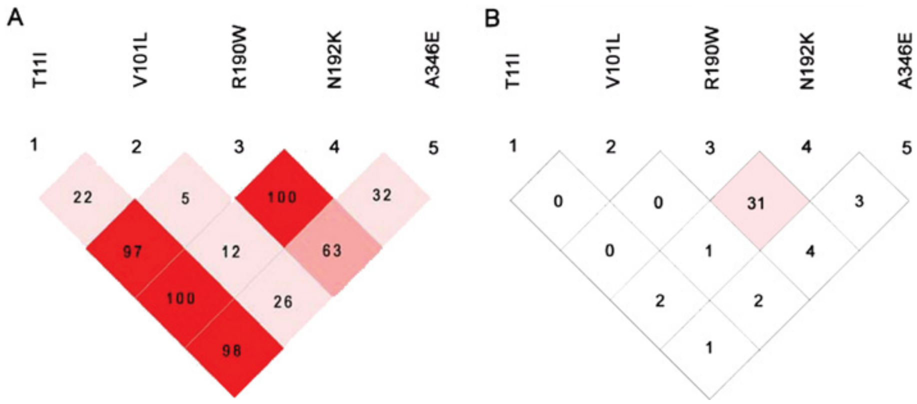

Figure S2 LD at human *FPR1*

The plot was generated with SNPs p.T11I (rs5030878), p.V101L (rs2070745), p.R190W (rs5030880), p.N192K (rs1042229) and p.E346A (rs867228) by SHEsis. Each diamond represents Lewontin's D' (**A**) or correlation coefficient r^2 (**B**). The darker the diamond, the greater the LD between the SNPs.

Received 8 December 2012/2 February 2013; accepted 4 February 2013
Published as BJ Immediate Publication 4 February 2013, doi:10.1042/BJ20121839

[1] To whom correspondence should be addressed (email wangmw@mail.shcnc.ac.cn).

© 2013 The Author(s)
The author(s) has paid for this article to be freely available under the terms of the Creative Commons Attribution Licence (CC-BY) (http://creativecommons.org/licenses/by/3.0/) which permits unrestricted use, distribution and reproduction in any medium, provided the original work is properly cited.

Biochemical Journal www.biochemj.org
